# 

**Published:** 1903-05

**Authors:** 


### CONTENTS.

ORIGINAL COMMUNICATIONS—

THE SIN OF SO-CALLED CONSERVATIVE MEDICAL TREATMENT IN DISEASES REQUIRING PROMPT SURGICAL INTERVENTION. By Floyd Wilcox McRae, M.D., Atlanta, Ga. .......... 61

THE TREATMENT OF CARCINOMA OF THE CERVIX. By Virgil O. Hardon, M.D., Atlanta, Ga .......... 66

SPINAL CURVATURE. By Theo. Toepel, M.D., Atlanta, Ga .......... 72

EDITORIAL NOTES AND COMMENTS—

THE INCREASE OF HOSPITALS IN ATLANTA .......... 77

THE COLUMBUS MEETING OF THE GEORGIA MEDICAL ASSOCIATION .......... 78

THE TYBEE LEPER .......... 79

NEWS AND NOTES .......... 81

CONTENTS CONTINUED .......... x

